# Development of a population‐level prediction model for intensive care unit (ICU) survivorship and mortality in older adults: A population‐based cohort study

**DOI:** 10.1002/hsr2.1634

**Published:** 2023-10-19

**Authors:** Sikandar H. Khan, Anthony J. Perkins, Mikita Fuchita, Emma Holler, Damaris Ortiz, Malaz Boustani, Babar A. Khan, Sujuan Gao

**Affiliations:** ^1^ Division of Pulmonary, Critical Care Sleep and Occupational Medicine Indianapolis Indiana USA; ^2^ Regenstrief Institute Indiana University Center for Aging Research Indianapolis Indiana USA; ^3^ Department of Medicine Indiana University School of Medicine Indianapolis Indiana USA; ^4^ Department of Biostatistics and Health Data Science Indiana University School of Medicine Indianapolis Indiana USA; ^5^ Department of Anesthesiology University of Colorado Anschutz Medical Campus Aurora Colorado USA; ^6^ Department of Epidemiology and Biostatistics Indiana University School of Public Health Bloomington Indiana USA; ^7^ Department of Surgery Indiana University School of Medicine Indianapolis Indiana USA; ^8^ Center for Health Innovation and Implementation Science Indiana University School of Medicine Indianapolis Indiana USA

**Keywords:** critical care outcomes, mortality, population health, risk

## Abstract

**Background and Aims:**

Given the growing utilization of critical care services by an aging population, development of population‐level risk models which predict intensive care unit (ICU) survivorship and mortality may offer advantages for researchers and health systems. Our objective was to develop a risk model for ICU survivorship and mortality among community dwelling older adults.

**Methods:**

This was a population‐based cohort study of 48,127 patients who were 50 years and older with at least one primary care visit between January 1, 2017, and December 31, 2017. We used electronic health record (EHR) data to identify variables predictive of ICU survivorship.

**Results:**

ICU admission and mortality within 2 years after index primary care visit date were used to divide patients into three groups of “alive without ICU admission”, “ICU survivors,” and “death.” Multinomial logistic regression was used to identify EHR predictive variables for the three patient outcomes. Cross‐validation by randomly splitting the data into derivation and validation data sets (60:40 split) was used to identify predictor variables and validate model performance using area under the receiver operating characteristics (AUC) curve. In our overall sample, 92.2% of patients were alive without ICU admission, 6.2% were admitted to the ICU at least once and survived, and 1.6% died. Greater deciles of age over 50 years, diagnoses of chronic obstructive pulmonary disorder or chronic heart failure, and laboratory abnormalities in alkaline phosphatase, hematocrit, and albumin contributed highest risk score weights for mortality. Risk scores derived from the model discriminated between patients that died versus remained alive without ICU admission (AUC = 0.858), and between ICU survivors versus alive without ICU admission (AUC = 0.765).

**Conclusion:**

Our risk scores provide a feasible and scalable tool for researchers and health systems to identify patient cohorts at increased risk for ICU admission and survivorship. Further studies are needed to prospectively validate the risk scores in other patient populations.

## Key Points

- Existing predictive models for critical illness often utilize clinical data from emergency room settings or assist in triage of patients admitted to the hospital. These predictive models do not differentiate outpatients who may develop critical illness in the future.
- Our study describes development of a predictive risk score to identify future predict intensive care unit (ICU) admission and ICU survival among community dwelling older adults seen for routine primary care.
- This risk prediction model performed well in identification of future ICU survivors, and may be a novel tool for researchers and health systems to recruit patients for clinical research.

## 1 INTRODUCTION

More than 5 million patients are admitted to intensive care units (ICUs) in the United States each year and utilization of critical care services is forecasted to increase given an aging population living longer and with more comorbidities. Despite advances in treatment of sepsis and acute respiratory failure, up to 29% of patients die during the ICU admission while another 15%−25% of older adults die within 30 days after ICU discharge.^1^, ^2^, ^3^, ^4^ Even survivors of critical illness experience cognitive, physical, and psychological impairments, collectively termed PostIntensive Care Syndrome (PICS).^5^, ^6^, ^7^, ^8^, ^9^ Given the morbidity associated with critical illness and PICS, novel approaches that can stratify the population by risk for critical illness are of growing scientific interest. Such population‐based models for ICU admission, mortality, and survival past the index admission^7^, ^10^, ^11^ could be used by researchers and health systems to design, study, and implement programs for patient populations at higher risk for critical illness and development of PICS.

Electronic health record (EHR) data offers the possibility of developing predictive, scalable, and generalizable models for prognostication.^12^ There have been recent advancements in the field of risk prediction models using EHR data to predict ICU mortality or identify community dwelling adults at risk for critical illness.^13^, ^14^, ^15^, ^16^, ^17^, ^18^, ^19^, ^20^, ^21^ However, many of these prediction tools include mortality within their definition of critical illness with limited discriminant ability to identify patients who will be ICU survivors.^14^, ^15^, ^22^, ^23^, ^24^ Predictive risk scores that can discriminate among these outcomes hold potential to improve current care pathways in two ways: (a) early identification of older adults in a community or health system at highest risk for ICU admission allowing recruitment and follow up in cohort studies; and, (b) development of novel health services programs and infrastructure by critical care stakeholders to advance the care for populations at risk for future critical illness and PICS. With the above goals in mind, we conducted this study using EHR data from a large cohort of older adults seen in the primary care setting to develop and validate a multivariable and scalable risk prediction tool for ICU survivorship and mortality. Our goal was to develop a population‐level prediction model to be utilized by researchers and hospital administrators.

## 2 METHODS

### 2.1 Study participants

This is a population‐based cohort study conducted at Indiana University School of Medicine. The study was approved by the institutional review board at Indiana University (IRB number: 13,488, Study Title: Predictive Models for ICU Admission in Older Adults, Approval Type: Exempt), and all study activities were conducted in accordance with the Declaration of Helsinki. We report our methods and results in accordance with the Transparent Reporting of a Multivariable Prediction Model for Individual Prognosis or Diagnosis (TRIPOD) reporting guidelines.^25^

The study cohort comprised patients aged 50 years or older who had at least one visit with a primary care provider (PCP) at either of two urban, academic, health care systems (Eskenazi Health and Indiana University Health) affiliated with Indiana University School of Medicine, in Indianapolis, Indiana, between January 1, 2017 and December 31, 2017. This date range was chosen so that patients' subsequent 2‐year outcomes in 2018−2019 could be used without the influence of the COVID‐19 pandemic. Both health systems provide care to a socioeconomically, racially, and geographically diverse patient population in the Indianapolis region. We chose to develop our prediction model in older adults (age 50 and older) given their increased rates of critical care utilization and high rates of PICS.

### 2.2 Data elements

We utilized the Indiana Network for Patient Care (INPC), a statewide EHR data warehouse, to identify patients meeting the eligibility criteria and perform the automated data extraction.^26^ INPC is the largest health information exchange in the United States with data comprising more than 13 million patients across the state of Indiana. For each patient included in the cohort, we defined an index date as the patient's last PCP visit date in 2017. If a patient had more than one PCP visit during the study year (2017), we used the last PCP visit as the index date. We then retrieved structured EHR data including demographics, medical history (ICD‐9 and ICD‐10 codes), laboratory results, medication orders, and health care utilization (outpatient clinic visits, emergency room visits, hospitalizations, and ICU admissions) from 2 years before index date. We collected ICU admission and mortality data for 2 years after index PCP visit.

We first converted all ICD‐9 codes into ICD‐10 codes using a SAS data set downloaded from the Center for Medicare and Medicaid website. In logistic regression models with many predictors, binary predictors with low frequencies may lead to problems with the convergence of the likelihood function and unstable parameter estimates. Therefore, common practice for conducting logistic models with a large number of predictors is to exclude binary variables with small event frequencies. To prevent a potential model fitting problem which could result in unstable parameter estimates, medical conditions with less than 2% prevalence in the overall sample were excluded, resulting in 211 unique ICD‐10 codes which were included in the development of our model (see Supporting Information: Table 1 for list of these ICD codes).^27^ We also included laboratory tests which are commonly obtained in the outpatient setting, such as complete blood count, comprehensive metabolic panel, lipids (total cholesterol, triglyceride, high‐density lipoprotein, and low‐density lipoprotein), and thyroid function (see Supporting Information: Table 2 for list of laboratory tests included in the model development). Laboratory values were dichotomized as normal or abnormal determined using published laboratory standards to minimize the potential influence of extreme values (see Supporting Information: Table 2). Patients who did not have a laboratory test within the 2‐years before the index visit were treated as having a normal value. We used the medication Generic Product Identifier code to classify medications into drug classes (see Supporting Information: Table 3 for list of medications included in the model development) and excluded medications with less than 2% prevalence in the overall sample.^28^

### 2.3 Outcome measures

We categorized patients in the cohort into three mutually exclusive outcome groups.

#### 2.3.1 Alive without ICU admission

Patients who survived the 2‐year follow‐up period after index PCP visit and were never admitted to the ICU.

#### 2.3.2 ICU survivor

Patients who had at least one ICU admission within 2 years of index PCP visit, and survived for at least 30 days after the first ICU discharge.

#### 2.3.3 Death

Patients who died within 2 years of index time, including patients who died without an ICU admission, those who died during the first ICU admission, or those who died within 30 days after discharge from the first ICU admission.

### 2.4 Analytic methods

#### 2.4.1 Model development

We compared patients' characteristics and medical conditions using analysis of variance for continuous measures and *χ* ^2^ tests for categorical measures among the three outcome categories using a 2‐sided *p* value of <0.05. Multinomial logistic models with the three outcomes categories described above were used to identify patients' characteristics, medical conditions, laboratory results and medication classes that were associated with the three patient groups. We chose this approach for model development as our goal was to create risk scores that are meaningful and straight‐forward to calculate, differentiating our model from machine learning prediction tools which are often too complex to interpret.^29^ Given the large number of potential variables contained in EHR data, we used forward model selection with Schwarz's Bayesian Criterion (SBC) as the model selection criterion. In each iteration of a forward model selection process, we evaluated the impact of adding one predictor variable to the model and select the predictor with the lowest SBC. The SBC penalizes models for their complexity to prevent overfitting.^30^, ^31^

To determine model validity, we performed cross‐validation by randomly splitting the data into a derivation data set and a validation data set (60:40 split) ten times. Predictor variables were retained in our model if they were selected in at least 50% of models from the derivation data set. Parameter estimates from selected predictor variables were averaged across all 10 cross‐validation models to create predictive risk scores for “death versus alive without ICU admission” and “ICU Survivors versus Alive without ICU Admission.” All analyses were performed using SAS v9.4.

#### 2.4.2 Comparing predictive performance with three previous EHR‐based risk scores

In the validation data sets, we compared our risk scores' predictive performance with three previous EHR‐based risk scores (Elder Risk Assessment [ERA], Multimorbidity Frailty Index [MFI], and the Hospital Frailty Risk Score [HFRS]).^24^, ^27^, ^32^ The ERA index was derived using data from the Mayo Clinic EHR to predict critical illness (defined as sepsis, need for mechanical ventilation, or death) within 2 years of the index outpatient visit. The MFI incorporated 32 deficits based on EHR using Taiwan's National Health Record for the prediction of ICU admission. The Hospital Frailty Score was developed using ICD‐10 codes that characterized frailty to predict mortality, prolonged hospital stay, and readmission among older adults. All risk scores were calculated in the validation data sets to measure model performance and calculate the average area under the Receiver Operator Characteristic (ROC) curves for ERA, MFI, HFRS and our two risk scores (the ICU Survivor Risk Score and the Mortality Risk Score).

## 3 RESULTS

We identified an overall sample of 48,127 patients who were aged 50 years and older and had at least one PCP visit in 2017. The study cohort was 60.8% female (29,251/48,127), 36.5% were 65 years or older (17,573/48,127), 62.3% white (29,995/48,127), 32.1% black (15,458/48,127), 5.7% Hispanic (2708/48,127), and 15.2% had Medicaid insurance (7299/48,127). During the 2‐year follow‐up period, 92.2% of patients were alive and never admitted to the ICU (44,370/48,127), 6.2% were admitted to the ICU at least once and survived (2994/48,127), and 1.6% died (763/48,127). Rates of demographics, as well as variables selected for the risk scores (ICD‐10 diagnoses, laboratory values, clinical testing, and health care utilization before the index primary care visit by the outcome groups) are shown in Supporting Information: Table 4. Among the three groups, ICU survivors had the highest rate of diabetes mellitus type II while patients that died had the highest rates of chronic obstructive pulmonary disease (COPD), congestive heart failure (CHF), and abnormal chest imaging (see Supporting Information: Table 4). ICD diagnoses for encounters for screening for malignant neoplasms were most frequent in patients who remained alive and were never admitted to the ICU. Characteristics of the predictor variables were similarly distributed between derivation (*n* = 29,042) and validation (*n* = 19,085) samples (see Supporting Information: Table 5).

### 3.1 Predictor variables associated with outcomes

Average parameter estimates and associated risk score weights for each of the retained predictors from the 10 testing data sets are shown in Table 1. We derived two risk scores: ICU Survivor Risk Score: score range 0−26, with higher scores indicating greater risk of ICU survivorship, and the Mortality Risk Score: score range 0−41, with higher scores indicating greater risk of death within 2 years.

**Table 1:** Results of multinomial logistic model comparing death and ICU survival to no events in 10 derivation samples.

Predictor Variables	# of models^a^	Parameter Estimates		Risk Score Weights	
		Death versus Alive Without ICU Admission	ICU Survival versus Alive Without ICU Admission	Death versus Alive Without ICU Admission	ICU Survivor versus Alive without ICU Admission
Age					
50−64 (reference)	−	−	−	0	0
65−74	10	0.53207	0.19248	3	1
75−84	10	1.11281	0.21419	6	1
85+	10	1.65922	0.24481	8	1
Abnormal Albumin	10	0.66821	0.31764	3	2
Abnormal Hematocrit	10	0.58149	0.48747	3	2
Abnormal Red Blood Cell Count	10	0.49476	0.38497	2	2
Elevated Alkaline Phosphatase	10	0.85576	0.3347	4	2
I50: Heart Failure	10	0.56241	0.3555	3	2
I73: Other peripheral vascular diseases	10	0.23695	0.5884	1	3
J44: Other chronic obstructive pulmonary disease	10	0.71458	0.53454	4	3
Z12: Encounter for screening for malignant neoplasms (absence)	10	−0.54646	−0.28371	3	1
Abnormal Creatinine	9	0.30474	0.31131	2	2
J30: Vasomotor and Allergic Rhinitis (absence)	6	−0.33159	−0.21872	2	1
Abnormal Sodium	5	0.13557	0.1503	1	1
Emergency Department Visit in Prior Year	5	0.07922	0.15119	0	1
E11: Diabetes mellitus type II	5	−0.0119	0.15424	0	1
I48: Atrial fibrillation and flutter	5	0.19716	0.19426	1	1
Z79: Long term (current) drug therapy	5	0.09732	0.14114	0	1

^a^ number of times a predictor was selected out of 10 testing data sets for cross validation.

In our models, greater deciles of age over 50, diagnoses of COPD and CHF, and laboratory abnormalities in serum alkaline phosphatase, hematocrit, and albumin contributed the highest risk score weights for mortality, while ICD‐10 diagnoses of encounter for screening for malignant neoplasms, and vasomotor or allergic rhinitis were associated with lower risk of death. In contrast, diagnoses of peripheral vascular disease and COPD were associated with ICU survival (see Table 1). Abnormal values for creatinine, red blood cell count, sodium, and diagnoses of atrial fibrillation/atrial flutter were retained predictors in risk scores for death and for ICU survivors.

### 3.2 Performance of predictive risk scores

We measured the predictive performance of our risk scores in ten validation samples (*n* = 19,085 in each sample). In Table 2, we present the average AUCs (and range) in overall performance and separately for three outcome groups. We also include mean AUCs for other electronic prediction tools (ERA, MFI, and HFRS) in Table 2.

**Table 2:** Average area under the curve (AUC) of predictive risk scores in 10 validation samples (*n* = 19,085 in each sample).

Scores	Overall	Death versus alive without ICU admission	ICU‐Survivor versuss alive without ICU admission	ICU survivor versus death
ERA	0.673 (0.662−0.680)	0.797 (0.772−0.813)	0.721 (0.713−0.729)	0.500
MFI	0.656 (0.651−0.667)	0.760 (0.744−0.783)	0.707 (0.699−0.718)	0.500
HFRS	0.633 (0.626−0.640)	0.725 (0.706−0.747)	0.673 (0.662−0.685)	0.500
Predictive Risk Score	0.715 (0.708−0.725)	0.854 (0.839−0.873)	0.766 (0.758−0.775)	0.527 (0.519−0.536)

*Note*: Minimum and maximum AUCs in the 10 validation samples are included in parentheses.

Abbreviations: ERA, Elder Risk Assessment; HFRS: Hospital Frailty Risk Score; MFI, Multimorbidity Frailty Index.

**Figure 1:**
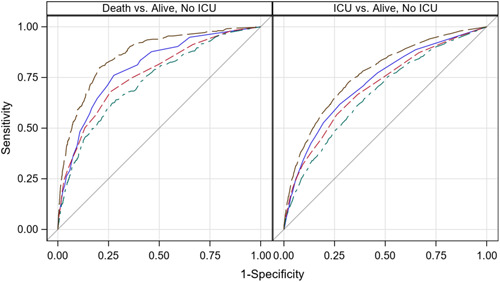
Receiver Operating Characteristic (ROC) Curves of Predictive Risk Scores Compared to Elder Risk Assessment (ERA), Multimorbidity Frailty Index (MFI), and Hospital Frailty Risk Score (HFRS) in one validation sample (*n* = 19,085). Elder Risk Assessment, ERA shown in blue. MFI shown in red. HFRS shown in green, Predictive Risk Score shown in brown.

Our predictive risk scores performed better than ERA, MFI, and HFRS in differentiating among any of the three possible outcomes (death vs. alive without ICU admission, ICU survivor vs. alive without ICU admission, and ICU survivor vs. death). In particular, our predictive risk scores had mean AUC of 0.766 in discriminating ICU survivors from those alive without ICU admission and mean AUC of 0.854 in predicting mortality versus alive without ICU admission. ROC curves for our predictive risk scores, ERA, MFI and HFRS in one validation sample are presented in Figure 1.

In Table 3, we provide positive predictive values (PPV) for the risk scores using suggested cut‐off points to categorize patients into high, medium, and low risk groups for ICU survivorship and death in one validation sample. Patients in the high‐risk group for ICU survivors (ICU survivor risk score ≥18) had the highest proportion of ICU survivorship (PPV = 33.8% compared to 18.1% in those with scores 11−17 and 4.2% in patients with score ≤10). Patients in the high‐risk group for death (Death risk score ≥22) also had the highest proportion of death (PPV = 13.9% compared to 5.7% in those with scores 16−21 and 0.8% in patients with score ≤15). We note in the overall cohort, rates of ICU survivorship and death are 6.2% and 1.6%, respectively.

**Table 3:** Numbers (proportions) of patients in each outcome group using cut‐off predictive risk scores, and positive predictive values, in one validation sample (*n* = 19,085).

Outcomes	Predictive risk score for ICU survivors		
	0−10 (*n* = 16,487)	11−17 (*n* = 2350)	18+ (*n* = 296)
	86.2%	12.3%	1.6%
Outcome, *n* (%)			
Alive without ICU admission	15,652 (94.9)	1803 (76.7)	157 (53.0)
Death	149 (0.9)	121 (5.2)	39 (13.2)
ICU Survivor	686 (4.2)	426 (18.1)	100 (33.8)

	Predictive risk score for death		
Outcomes	0−15 (*n* = 16847)	16−21 (*n* = 1704)	22+ (*n* = 582)
	88.0%	8.9%	3.0%
Outcome, *n* (%)			
Alive without ICU admission	15948 (94.7)	1319 (77.4)	345 (59.3)
Death	131 (0.8)	97 (5.7)	81 (13.9)
ICU Survivor	768 (4.6)	288 (16.9)	156 (26.8)

Abbreviation: ICU, intensive care unit.

## 4 DISCUSSION

We developed two EHR‐based predictive risk scores (ICU Survivor Risk Score and Mortality Risk Score) to identify community dwelling older adults at risk for ICU admission/survivorship or death. In contrast with other recent studies, we categorized ICU survivorship as an independent outcome group with the objective of identifying older adults in the population at risk for ICU admission. Our models are intended to assist hospitals, health systems, and researchers identify cohorts of patients at higher risk for critical illness survivorship (i.e., patients at risk for PICS) and mortality, rather than for bedside clinical prognostication at the individual patient level. We found that our model's risk scores outperformed previously validated EHR‐based risk predictions in ICU survival (AUC 0.765) and death (AUC 0.858) within 2‐years.

In recent years, a growing number of models that predict patient‐level outcomes have been published. These models have improved our ability to predict need for ICU admission when evaluating patients in the emergency department or hospital ward, ICU mortality for patients being admitted to the hospital or the ICU, and for risk of readmission to the ICU for those being discharged.^33^, ^34^, ^35^, ^36^ The goals of our predictive models were instead to utilize EHR data in the pre‐ICU phase to identify groups of patients at increased risk for future ICU admission and survival within 2 years. In this paper, we present the development of two practical and scalable risk scores that focus on earlier detection of patients at risk for poor outcomes, that is, in community dwelling adults in the “pre‐ICU phase.”

In the process of development of the risk scores, we also identified the variables associated with increased risk of mortality. These predictors included older age, diagnoses of COPD, CHF, and laboratory abnormalities in multiple organ systems (such as liver, kidney, and blood).^13^, ^14^ By their design, however, prior studies relied on in‐hospital or in‐ICU clinical data, whereas our study includes variables that may be predictive of mortality even when measured in the outpatient, pre‐ICU phases of care.

We also identified variables in our risk score which were associated with ICU survival. Diagnoses of COPD, CHF, and PVD were all associated with ICU admission/survival. In addition, diagnoses of vasomotor/allergic rhinitis had lower risk of mortality or ICU admission/survival, perhaps due to increased health care contact. While not inferred to be causal or intended to spur clinical action on an individual patient level, our results suggest additional opportunities for early identification of patients and mechanisms associated with critical illness that may warrant further study. It is worth noting that our predictive risk scores and the ERA both included age as a predictor variable. While our predictive scores provided the best predictive accuracy in terms of AUC, ERA provided the second‐best prediction among all risk scores compared. While every decade increase in age drastically increased mortality risk, the risk for ICU admission/survivorship appears constant for those age 65 or older, a notable distinction between risk prediction for ICU survivorship and mortality.

The ability of our scores to identify patients at risk for admission to the ICU, and mortality, may offer advantages for researchers. The risk scores may create opportunities for researchers to identify, screen, recruit and measure long‐term outcome trajectories within this medically complex patient population, as well as involve patients in the design of novel pre‐, intra‐, and post‐ICU interventions focused on recovery and resilience.^37^

Our study has several strengths. We utilized a comprehensive medical record database with diverse socioeconomic and racial representation. Second, our study's large sample size allowed the use of cross‐validation for the development and testing of the predictive models so that both testing and validation samples are sufficiently large to provide stable estimates. Third, model selection was based on collective model performance criteria instead of significance of single variables. Finally, we compared our predictive models to several current EHR‐based risk scores to demonstrate the superior performance of our model prediction.

Our study also has limitations associated with the limitation of EHR data. First, the EHR data did not include some variables known to be associated with mortality such as socioeconomic status (including the level of education), nutrition status or physical activity levels. Second, our model is developed to predict outcomes among older adults, and therefore its validity in younger adults at risk for PICS is not yet known. Third, as our inclusion criteria required an index PCP visit, how well the model identifies patients who were never seen in primary care before critical illness is not known. Finally, our model's limited performance in differentiating between the groups of patients who will survive the ICU versus those who die suggests additional data input is needed for improving predictive accuracy between these two outcome groups.

In summary, we developed and validated risk scores to predict outcomes in older adults. Our models perform well in identification of patients at risk for death, and those who will survive after ICU admission. The future scope of our work includes confirmation of the Predictive Risk Scores in large, prospective cohort studies. Implementation of the risk scores within population health settings may enable early identification of older adults at risk for critical illness, allowing researchers and health systems to design and test novel interventions to optimize post‐ICU recovery.

## AUTHOR CONTRIBUTIONS

**Sikandar H. Khan**: Investigation; writing—original draft; writing—review and editing. **Anthony J. Perkins**: Data curation; formal analysis; writing—review and editing. **Mikita Fuchita**: Writing—review and editing. **Emma Holler**: Writing—review and editing. **Damaris Ortiz**: Writing—review and editing. **Malaz Boustani**: Writing—review and editing. **Babar A. Khan**: Writing—review and editing. **Sujuan Gao**: Formal analysis; writing—review and editing. All authors have read and approved the final version of the manuscript.

## CONFLICT OF INTEREST STATEMENT

The authors declare no conflict of interest.

## TRANSPARENCY STATEMENT

The lead author Sikandar H. Khan, Sikandar H. Khan affirms that this manuscript is an honest, accurate, and transparent account of the study being reported; that no important aspects of the study have been omitted; and that any discrepancies from the study as planned (and, if relevant, registered) have been explained.

## Supporting information

Supporting information.

## Data Availability

Individual participant data is not available without permission from the participating health systems. Sikandar Khan had full access to all of the data in this study and takes complete responsibility for the integrity of the data and the accuracy of the data analysis.
