# Supplementary material for: Development of a population‐level prediction model for intensive care unit (ICU) survivorship and mortality in older adults: A population‐based cohort study
Source: Health Sci Rep. 2023 Oct 19;6(10):e1634. doi: 10.1002/hsr2.1634 (PMC10587446; doi:10.1002/hsr2.1634)
Supplement: Supplementary file 1 — Supporting information. [file HSR2-6-e1634-s001.docx]

**Supplementary Online Content**

**Supplementary Table S1.** List of ICD-10 codes and associated diagnoses included in the model selection

**Supplementary Table S2**. Laboratory tests and cut-off values included in the model selection

**Supplementary Table S3**. List of medications and drug classes included in the model selection

**Supplementary Table S4.** Patient characteristics by outcome groups (Alive without ICU Admission, ICU Survivor, Death)

**Supplemental Table S5.** Patient characteristics and predictor variables by one cross-validation sample

**Supplementary Table S1. List of ICD-10 codes and associated diagnoses included in the model selection**

| **International Classification of Disease (ICD)-10 Code and Diagnosis** |
| --- |
| B18 - Chronic viral hepatitis |
| B35 - Dermatophytosis |
| B37 - Candidiasis |
| B96 - Other bacterial agents as the cause of diseases classified elsewhere |
| C50 - Malignant neoplasm of breast |
| D12 - Benign neoplasm of colon, rectum, anus and anal canal |
| D48 - Neoplasm of uncertain behavior of other and unspecified sites |
| D50 - Iron deficiency anemia |
| D64 - Other anemias |
| D69 - Purpura and other hemorrhagic conditions |
| D72 - Other disorders of white blood cells |
| E03 - Other hypothyroidism |
| E04 - Other nontoxic goiter |
| E10 - Type 1 diabetes mellitus |
| E11 - Type 2 diabetes mellitus |
| E13 - Other specified diabetes mellitus |
| E53 - Deficiency of other B group vitamins |
| E55 - Vitamin D deficiency |
| E66 - Overweight and obesity |
| E78 - Disorders of lipoprotein metabolism and other lipidemias |
| E83 - Disorders of mineral metabolism |
| E86 - Volume depletion |
| E87 - Other disorders of fluid, electrolyte and acid-base balance |
| F10 - Alcohol related disorders |
| F17 - Nicotine dependence |
| F31 - Bipolar disorder |
| F32 - Major depressive disorder, single episode |
| F33 - Major depressive disorder, recurrent |
| F34 - Persistent mood [affective] disorders |
| F41 - Other anxiety disorders |
| F43 - Reaction to severe stress, and adjustment disorders |
| F51 - Sleep disorders not due to a substance or known physiological condition |
| G25 - Other extrapyramidal and movement disorders |
| G31 - Other degenerative diseases of nervous system, not elsewhere classified |
| G43 - Migraine |
| G44 - Other headache syndromes |
| G47 - Sleep disorders |
| G56 - Mononeuropathies of upper limb |
| G62 - Other and unspecified polyneuropathies |
| G89 - Pain, not elsewhere classified |
| G93 - Other disorders of brain |
| H04 - Disorders of lacrimal system |
| H10 - Conjunctivitis |
| H25 - Age-related cataract |
| H26 - Other cataract |
| H35 - Other retinal disorders |
| H40 - Glaucoma |
| H52 - Disorders of refraction and accommodation |
| H53 - Visual disturbances |
| H61 - Other disorders of external ear |
| H66 - Suppurative and unspecified otitis media |
| H81 - Disorders of vestibular function |
| H90 - Conductive and sensorineural hearing loss |
| H91 - Other and unspecified hearing loss |
| H92 - Otalgia and effusion of ear |
| H93 - Other disorders of ear, not elsewhere classified |
| I10 - Essential (primary) hypertension |
| I12 - Hypertensive chronic kidney disease |
| I21 - ST elevation (STEMI) and non-ST elevation (NSTEMI) myocardial infarction |
| I25 - Chronic ischemic heart disease |
| I48 - Atrial fibrillation and flutter |
| I49 - Other cardiac arrhythmias |
| I50 - Heart failure |
| I51 - Complications and ill-defined descriptions of heart disease |
| I63 - Cerebral infarction |
| I65 - Occlusion and stenosis of precerebral arteries, not resulting in cerebral infarction |
| I70 - Atherosclerosis |
| I73 - Other peripheral vascular diseases |
| I82 - Other venous embolism and thrombosis |
| I87 - Other disorders of veins |
| I95 - Hypotension |
| J01 - Acute sinusitis |
| J02 - Acute pharyngitis |
| J06 - Acute upper respiratory infections of multiple and unspecified sites |
| J18 - Pneumonia, unspecified organism |
| J20 - Acute bronchitis |
| J30 - Vasomotor and allergic rhinitis |
| J32 - Chronic sinusitis |
| J34 - Other and unspecified disorders of nose and nasal sinuses |
| J40 - Bronchitis, not specified as acute or chronic |
| J43 - Emphysema |
| J44 - Other chronic obstructive pulmonary disease |
| J45 - Asthma |
| J96 - Respiratory failure, not elsewhere classified |
| J98 - Other respiratory disorders |
| K21 - Gastro-esophageal reflux disease |
| K52 - Other and unspecified noninfective gastroenteritis and colitis |
| K57 - Diverticular disease of intestine |
| K58 - Irritable bowel syndrome |
| K59 - Other functional intestinal disorders |
| K62 - Other diseases of anus and rectum |
| K63 - Other diseases of intestine |
| K64 - Hemorrhoids and perianal venous thrombosis |
| K76 - Other diseases of liver |
| K92 - Other diseases of digestive system |
| L02 - Cutaneous abscess, furuncle and carbuncle |
| L03 - Cellulitis and acute lymphangitis |
| L29 - Pruritus |
| L30 - Other and unspecified dermatitis |
| L57 - Skin changes due to chronic exposure to nonionizing radiation |
| L60 - Nail disorders |
| L82 - Seborrheic keratosis |
| L84 - Corns and callosities |
| L85 - Other epidermal thickening |
| L98 - Other disorders of skin and subcutaneous tissue, not elsewhere classified |
| M10 - Gout |
| M12 - Other and unspecified arthropathy |
| M15 - Polyosteoarthritis |
| M16 - Osteoarthritis of hip |
| M17 - Osteoarthritis of knee |
| M19 - Other and unspecified osteoarthritis |
| M20 - Acquired deformities of fingers and toes |
| M21 - Other acquired deformities of limbs |
| M25 - Other joint disorder, not elsewhere classified |
| M43 - Other deforming dorsopathies |
| M47 - Spondylosis |
| M48 - Other spondylopathies |
| M50 - Cervical disc disorders |
| M51 - Thoracic, thoracolumbar, and lumbosacral intervertebral disc disorders |
| M53 - Other and unspecified dorsopathies, not elsewhere classified |
| M54 - Dorsalgia |
| M62 - Other disorders of muscle |
| M65 - Synovitis and tenosynovitis |
| M70 - Soft tissue disorders related to use, overuse and pressure |
| M72 - Fibroblastic disorders |
| M75 - Shoulder lesions |
| M77 - Other enthesopathies |
| M79 - Other and unspecified soft tissue disorders, not elsewhere classified |
| M81 - Osteoporosis without current pathological fracture |
| M85 - Other disorders of bone density and structure |
| M89 - Other disorders of bone |
| N17 - Acute kidney failure |
| N18 - Chronic kidney disease (CKD) |
| N20 - Calculus of kidney and ureter |
| N28 - Other disorders of kidney and ureter, not elsewhere classified |
| N30 - Cystitis |
| N39 - Other disorders of urinary system |
| N40 - Enlarged prostate |
| N52 - Male erectile dysfunction |
| N63 - Unspecified lump in breast |
| N64 - Other disorders of breast |
| N76 - Other inflammation of vagina and vulva |
| N95 - Menopausal and other perimenopausal disorders |
| R00 - Abnormalities of heart beat |
| R03 - Abnormal blood-pressure reading, without diagnosis |
| R05 - Cough |
| R06 - Abnormalities of breathing |
| R07 - Pain in throat and chest |
| R09 - Other symptoms and signs involving the circulatory and respiratory system |
| R10 - Abdominal and pelvic pain |
| R11 - Nausea and vomiting |
| R13 - Aphagia and dysphagia |
| R19 - Other symptoms and signs involving the digestive system and abdomen |
| R20 - Disturbances of skin sensation |
| R21 - Rash and other nonspecific skin eruption |
| R22 - Localized swelling, mass and lump of skin and subcutaneous tissue |
| R23 - Other skin changes |
| R25 - Abnormal involuntary movements |
| R26 - Abnormalities of gait and mobility |
| R29 - Other symptoms and signs involving the nervous and musculoskeletal systems |
| R30 - Pain associated with micturition |
| R31 - Hematuria |
| R32 - Unspecified urinary incontinence |
| R35 - Polyuria |
| R39 - Other and unspecified symptoms and signs involving the genitourinary system |
| R41 - Other symptoms and signs involving cognitive functions and awareness |
| R42 - Dizziness and giddiness |
| R50 - Fever of other and unknown origin |
| R51 - Headache |
| R52 - Pain, unspecified |
| R53 - Malaise and fatigue |
| R55 - Syncope and collapse |
| R60 - Edema, not elsewhere classified |
| R63 - Symptoms and signs concerning food and fluid intake |
| R68 - Other general symptoms and signs |
| R73 - Elevated blood glucose level |
| R74 - Abnormal serum enzyme levels |
| R79 - Other abnormal findings of blood chemistry |
| R91 - Abnormal findings on diagnostic imaging of lung |
| R92 - Abnormal and inconclusive findings on diagnostic imaging of breast |
| R93 - Abnormal findings on diagnostic imaging of other body structures |
| R94 - Abnormal results of function studies |
| T14 - Injury of unspecified body region |
| W19 - Unspecified fall |
| Z00 - Encounter for general examination without complaint, suspected or reported diagnosis |
| Z01 - Encounter for other special examination without complaint, suspected or reported diagnosis |
| Z09 - Encounter for follow-up examination after completed treatment for conditions other than malignant neoplasm |
| Z11 - Encounter for screening for infectious and parasitic diseases |
| Z12 - Encounter for screening for malignant neoplasms |
| Z13 - Encounter for screening for other diseases and disorders |
| Z23 - Encounter for immunization |
| Z48 - Encounter for other postprocedural aftercare |
| Z51 - Encounter for other aftercare |
| Z53 - Persons encountering health services for specific procedures and treatment, not carried out |
| Z68 - Body mass index [BMI] |
| Z71 - Persons encountering health services for other counseling and medical advice, not elsewhere classified |
| Z72 - Problems related to lifestyle |
| Z74 - Problems related to care provider dependency |
| Z76 - Persons encountering health services in other circumstances |
| Z78 - Other specified health status |
| Z79 - Long term (current) drug therapy |
| Z80 - Family history of primary malignant neoplasm |
| Z85 - Personal history of malignant neoplasm |
| Z86 - Personal history of certain other diseases |
| Z87 - Personal history of other diseases and conditions |
| Z88 - Allergy status to drugs, medicaments and biological substances |
| Z90 - Acquired absence of organs, not elsewhere classified |
| Z91 - Personal risk factors, not elsewhere classified |
| Z95 - Presence of cardiac and vascular implants and grafts |
| Z96 - Presence of other functional implants |
| Z98 - Other postprocedural states |

**Supplementary Table S2. Laboratory tests and cut-off values included in the model selection.**

| **Laboratory Test and Units** | **Normal Range (or abnormal value cutoff)** |
| --- | --- |
| Albumin g/dL | 3.4 - 5.4 |
| Alkaline Phosphatase U/L | 40 - 147 |
| Alanine Transaminase (ALT) U/L | 7 - 55 |
| Aspartate Transferase (AST) U/L | 8 - 48 |
| Bicarb mEq/L | 23 – 29 |
| Bilirubin mg/dL | > 1.2 |
| Blood Urea Nitrogen (BUN) mg/dL | 6 - 24 |
| Calcium mg/dL | 8.5 - 10.2 |
| Cholesterol mg/dL | > 200 |
| Chloride mEq/L | 96 - 106 |
| Creatinine mg/dL | Males: 0.74 -1.35  Females: 0.59 - 1.04 |
| Glucose mg/dL | 2 different cutoffs: > 100 and >140 |
| Hematocrit % | Males: 38.3 - 48.6  Females: 35.5 - 44.9 |
| Hemoglobin g/dL | Males: 13.2 - 16.6  Females: 11.6 - 15.0 |
| High Density Lipoprotein (HDL) mg/dL | Males: < 40  Females: < 50 |
|  |  |
| Low Density Lipoprotein (LDL) mg/dL | 2 different cutoffs: >100 and >130 |
| Platelet k/uL | 150-450 |
| Potassium mmol/L | 3.6 -5.2 |
| Protein g/dL | 6.4 - 8.3 |
| Red Blood Cell million/mcL | Males: 4.7 - 6.1  Females: 4.2 - 5.4 |
| Sodium mEq/L | 135 - 150 |
| Thyroid Stimulating Hormone U/I | 0.4 – 4 |
| White Blood Cell per uL | 4000 - 11000 |

**Supplementary Table S3. List of medications and drug classes included in the model selection**

| **Type of Medication(s)** | **Class of Drug** | **Drug Subclass** |
| --- | --- | --- |
| PENICILLINS | Aminopenicillins | Aminopenicillins |
| PENICILLINS | Penicillin Combinations | Penicillin Combinations |
| CEPHALOSPORINS | Cephalosporins - 1st Generation | Cephalosporins - 1st Generation |
| MACROLIDES | Azithromycin | Azithromycin |
| TETRACYCLINES | Tetracyclines | Tetracyclines |
| FLUOROQUINOLONES | Fluoroquinolones | Fluoroquinolones |
| ANTIFUNGALS | Imidazole-Related Antifungals | Imidazoles |
| ANTIVIRALS | Herpes Agents | Herpes Agents - Purine Analogues |
| ANTI-INFECTIVE AGENTS - MISC. | Anti-infective Agents - Misc. | Anti-infective Agents - Misc. |
| ANTI-INFECTIVE AGENTS - MISC. | Lincosamides | Lincosamides |
| ANTI-INFECTIVE AGENTS - MISC. | Urinary Anti-infectives | Urinary Anti-infectives |
| ANTI-INFECTIVE AGENTS - MISC. | Anti-infective Misc. - Combinations | Anti-infective Misc. - Combinations |
| VACCINES | Viral Vaccines | Viral Vaccines |
| CORTICOSTEROIDS | Glucocorticosteroids | Glucocorticosteroids |
| ANTIDIABETICS | Insulin | Mixed Insulin |
| ANTIDIABETICS | Sulfonylureas | Sulfonylureas |
| ANTIDIABETICS | Biguanides | Biguanides |
| THYROID AGENTS | Thyroid Hormones | Thyroid Hormones |
| ANTIANGINAL AGENTS | Nitrates | Nitrates |
| BETA BLOCKERS | Beta Blockers Cardio-Selective | Beta Blockers Cardio-Selective |
| BETA BLOCKERS | Alpha-Beta Blockers | Alpha-Beta Blockers |
| CALCIUM CHANNEL BLOCKERS | Calcium Channel Blockers | Calcium Channel Blockers |
| ANTIHYPERTENSIVES | ACE Inhibitors | ACE Inhibitors |
| ANTIHYPERTENSIVES | Angiotensin II Receptor Antagonists | Angiotensin II Receptor Antagonists |
| ANTIHYPERTENSIVES | Antiadrenergic Antihypertensives | Antiadrenergics - Centrally Acting |
| ANTIHYPERTENSIVES | Antihypertensive Combinations | Reserpine Combinations |
| DIURETICS | Loop Diuretics | Loop Diuretics |
| DIURETICS | Potassium Sparing Diuretics | Potassium Sparing Diuretics |
| DIURETICS | Thiazides and Thiazide-Like Diuretics | Thiazides and Thiazide-Like Diuretics |
| DIURETICS | Diuretic Combinations | Diuretic Combinations |
| ANTIHYPERLIPIDEMICS | HMG CoA Reductase Inhibitors | HMG CoA Reductase Inhibitors |
| NASAL AGENTS - SYSTEMIC AND TOPICAL | Nasal Steroids | Nasal Steroids |
| COUGH/COLD/ALLERGY | Antitussives | Antitussive - Opioid |
| COUGH/COLD/ALLERGY | Cough/Cold/Allergy Combinations | Decongestant-Analgesic |
| ANTIASTHMATIC AND BRONCHODILATOR AGENTS | Bronchodilators - Anticholinergics | Bronchodilators - Anticholinergics |
| ANTIASTHMATIC AND BRONCHODILATOR AGENTS | Sympathomimetics | Beta Adrenergics |
| ANTIASTHMATIC AND BRONCHODILATOR AGENTS | Leukotriene Modulators | 5-Lipoxygenase Inhibitors |
| LAXATIVES | Laxatives - Miscellaneous | Laxatives - Miscellaneous |
| LAXATIVES | Laxative Combinations | Laxative Combinations |
| ULCER DRUGS/ANTISPASMODICS/ANTICHOLINERGICS | H-2 Antagonists | H-2 Antagonists |
| ULCER DRUGS/ANTISPASMODICS/ANTICHOLINERGICS | Proton Pump Inhibitors | Proton Pump Inhibitors |
| ANTIEMETICS | Antiemetics - Anticholinergic | Antiemetics - Anticholinergic |
| ANTIEMETICS | 5-HT3 Receptor Antagonists | 5-HT3 Receptor Antagonists |
| URINARY ANTISPASMODICS | Urinary Antispasmodic - Antimuscarinics (Anticholinergic) | Urinary Antispasmodic - Antimuscarinic (Anticholinergic) |
| GENITOURINARY AGENTS - MISCELLANEOUS | Prostatic Hypertrophy Agents | 5-Alpha Reductase Inhibitors |
| ANTIANXIETY AGENTS | Benzodiazepines | Benzodiazepines |
| ANTIANXIETY AGENTS | Antianxiety Agents - Misc. | Antianxiety Agents - Misc. |
| ANTIDEPRESSANTS | Serotonin Modulators | Serotonin Modulators |
| ANTIDEPRESSANTS | Selective Serotonin Reuptake Inhibitors (SSRIs) | Selective Serotonin Reuptake Inhibitors (SSRIs) |
| ANTIDEPRESSANTS | Serotonin-Norepinephrine Reuptake Inhibitors (SNRIs) | Serotonin-Norepinephrine Reuptake Inhibitors (SNRIs) |
| ANTIDEPRESSANTS | Tricyclic Agents | Tricyclic Agents |
| ANTIDEPRESSANTS | Antidepressants - Misc. | Antidepressants - Misc. |
| HYPNOTICS/SEDATIVES/SLEEP DISORDER AGENTS | Non-Barbiturate Hypnotics | Non-Barbiturate Hypnotics |
| PSYCHOTHERAPEUTIC AND NEUROLOGICAL AGENTS - MISC. | Smoking Deterrents | Smoking Deterrents |
| ANALGESICS - NonNarcotic | Salicylates | Salicylates |
| ANALGESICS - OPIOID | Opioid Agonists | Opioid Agonists |
| ANALGESICS - OPIOID | Opioid Combinations | Opioid Combinations |
| ANALGESICS - ANTI-INFLAMMATORY | Nonsteroidal Anti-inflammatory Agents (NSAIDs) | Nonsteroidal Anti-inflammatory Agents (NSAIDs) |
| GOUT AGENTS | Gout Agents | Gout Agents |
| ANTICONVULSANTS | Anticonvulsants - Misc. | Anticonvulsants - Misc. |
| MUSCULOSKELETAL THERAPY AGENTS | Central Muscle Relaxants | Central Muscle Relaxants |
| MINERALS & ELECTROLYTES | Potassium | Potassium |
| ANTICOAGULANTS | Coumarin Anticoagulants | Coumarin Anticoagulants |
| HEMATOLOGICAL AGENTS - MISC. | Platelet Aggregation Inhibitors | Platelet Aggregation Inhibitors |
| OPHTHALMIC AGENTS | Ophthalmic Anti-infectives | Ophthalmic Antibiotics |
| OPHTHALMIC AGENTS | Ophthalmic Steroids | Ophthalmic Steroids |
| DERMATOLOGICALS | Antibiotics - Topical | Antibiotics - Topical |
| DERMATOLOGICALS | Antifungals - Topical | Antifungals - Topical |
| DERMATOLOGICALS | Anti-inflammatory Agents - Topical | Anti-inflammatory Agents - Topical |
| DERMATOLOGICALS | Corticosteroids - Topical | Corticosteroids - Topical |
| DIAGNOSTIC PRODUCTS | Diagnostic Tests | Diagnostic Tests |
| MEDICAL DEVICES AND SUPPLIES | Parenteral Therapy Supplies | Parenteral Therapy Supplies |
| MEDICAL DEVICES AND SUPPLIES | Diabetic Supplies | Insulin Administration Supplies |

**Supplementary Table S4. Patient characteristics by outcome groups (Alive without ICU Admission, ICU Survivor, Death)**

| **Variable** | **Overall**  **(n=48127)** | **Alive Without ICU Admission**  **(n=44370)** | **ICU Survivor**  **(n=2994)** | **Death**  **(n=763)** | **P-value** |
| --- | --- | --- | --- | --- | --- |
| **Age** | | | | | <0.001 |
| 50-64 (reference) | 30554 (63.5) | 28738 (64.8) | 1551 (51.8) | 265 (34.7) |  |
| 65-74 | 10817 (22.5) | 9821 (22.1) | 805 (26.9) | 191 (25.0) |  |
| 75-84 | 4913 (10.2) | 4283 (9.7) | 452 (15.1) | 178 (23.3) |  |
| 85+ | 1843 (3.8) | 1528 (3.4) | 186 (6.2) | 129 (16.9) |  |
| Female | 29251 (60.8) | 27276 (61.5) | 1571 (52.5) | 404 (53.0) | <0.001 |
| **Race (n=47037)** | | | | | <0.001 |
| White | 29995 (63.8) | 27732 (64.0) | 1795 (60.6) | 468 (61.9) |  |
| Black | 15458 (32.9) | 14084 (32.5) | 1103 (37.2) | 271 (35.8) |  |
| Other | 1584 (3.4) | 1503 (3.5) | 64 (2.2) | 17 (2.3) |  |
| **Ethnicity (n=47669)** | | | | | <0.001 |
| Hispanic | 2708 (5.7) | 2559 (5.8) | 122 (4.1) | 27 (3.6) |  |
| **Insurance (n=44426)** | | | | | <0.001 |
| Medicare | 18302 (41.2) | 16209 (39.7) | 1658 (58.0) | 435 (58.8) |  |
| Medicaid | 7299 (16.4) | 6719 (16.5) | 455 (15.9) | 125(16.9) |  |
| Commercial | 14754 (33.2) | 14107 (34.6) | 525 (18.4) | 122 (16.5) |  |
| Self-Pay | 1982 (4.5) | 1798 (4.4) | 135 (4.7) | 49 (6.6) |  |
| Other | 2089 (4.7) | 1993 (4.9) | 87 (3.0) | 9 (1.2) |  |
| **Diagnoses (ICD-10)** | | | | | |
| Z12: Encounter for screening for malignant neoplasms | 25068 (52.1) | 23576 (53.1) | 1262 (42.2) | 230 (30.1) | <0.001 |
| E11: Diabetes mellitus type II | 15277 (31.7) | 13564 (30.6) | 1391 (46.5) | 322 (42.2) | <0.001 |
| Z79: Long term (current) drug therapy | 14226 (29.6) | 12316 (27.8) | 1510 (50.4) | 400 (52.4) | <0.001 |
| J44: Other chronic obstructive pulmonary disease | 6034 (12.5) | 4892 (11.0) | 880 (29.4) | 262 (34.3) | <0.001 |
| J30: Vasomotor and Allergic Rhinitis | 5757 (12.0) | 5411 (12.2) | 283 (95.) | 63 (8.3) | <0.001 |
| I48: Atrial fibrillation and flutter | 2803 (5.8) | 2167 (4.9) | 475 (15.9) | 161 (21.1) | <0.001 |
| I50: Heart Failure | 3759 (7.8) | 2786 (6.3) | 720 (24.1) | 253 (33.2) | <0.001 |
| I73: Other peripheral vascular diseases | 2140 (4.4) | 1655 (3.7) | 389 (13.0) | 96 (12.6) | <0.001 |
| **Laboratory Abnormalities (Blood)** | | | | | |
| Red Blood Cell Count | 18345 (38.1) | 15772 (35.6) | 1994 (66.6) | 579 (75.9) | <0.001 |
| Creatinine | 15549 (32.3) | 13258 (29.9) | 1701 (59.5) | 510 (66.8) | <0.001 |
| Sodium | 8318 (15.3) | 6863 (15.5) | 1122 (37.5) | 333 (43.6) | <0.001 |
| Hematocrit | 5824 (12.1) | 4544 (10.2) | 990 (33.1) | 290 (38.0) | <0.001 |
| Albumin | 5789 (12.0) | 4440 (10.0) | 1001 (33.4) | 348 (45.6) | <0.001 |
| Alkaline Phosphatase (elevated) | 2329 (4.8) | 1788 (4.0) | 384 (12.8) | 157 (20.6) | <0.001 |
| Bicarbonate | 1201 (2.5) | 756 (1.7) | 358 (12.0) | 87 (11.4) | <0.001 |
| **Clinical Testing Performed** | | | | | |
| Electrocardiogram prior year | 13425 (27.9) | 11427 (25.8) | 1560 (52.1) | 438 (57.4) | <0.001 |
| **Healthcare Utilization** | | | | | |
| Emergency Department Visit in the Year Prior to Primary Care Visit | 17304 (36.0) | 15119 (34.1) | 1731 (57.8) | 454 (59.5) | <0.001 |

Data reported as n (%) unless otherwise specified. ICD: International Classification of Diseases.

**Supplemental Table S5**. **Patient characteristics and predictor variables by one cross-validation sample**

| **Characteristics** | **Overall**  **(n=48127)** | **Derivation**  **(n=28994)** | **Validation**  **(n=19133)** |  |
| --- | --- | --- | --- | --- |
| **Outcome** | | | |  |
| Alive, never admitted to ICU | 44370 (92.2) | 26758 (92.3) | 17612 (92.1) |  |
| Alive, admitted to the ICU | 2994 (6.2) | 1782 (6.2) | 1212 (6.3) |  |
| Death | 763 (1.6) | 454 (1.6) | 309 (1.6) |  |
| **Age** | | | |  |
| 50-64 | 30554 (63.5) | 18425 (63.6) | 12129 (63.4) |  |
| 65-74 | 10817 (22.5) | 6498(22.4) | 4319 (22.6) |  |
| 75-84 | 4913 (10.2) | 2971 (10.2) | 1942 (10.2) |  |
| 85+ | 1843 (3.8) | 1100 (3.8) | 743 (3.9) |  |
| Female | 29251 (60.8) | 17647 (60.9) | 11604 (60.7) |  |
| **Race** |  |  |  |  |
| White | 29995 (63.8) | 18062 (63.7) | 11933 (63.8) |  |
| Black | 15458 (32.9) | 9334 (32.9) | 6124 (32.8) |  |
| Other | 1584 (3.4) | 945 (3.3) | 639 (3.4) |  |
| **Ethnicity** |  |  |  |  |
| Hispanic | 2708 (5.7) | 1622 (5.6) | 1086 (5.7) |  |
| **Insurance** |  |  |  |  |
| Medicare | 18302 (41.2) | 11067 (41.4) | 7235 (41.0) |  |
| Medicaid | 7299 (16.4) | 4334 (16.2) | 2965 (16.8) |  |
| Commercial | 14754 (33.2) | 8874 (33.2) | 5880 (33.3) |  |
| Self-Pay | 1982 (4.5) | 1194 (4.5) | 788 (4.5) |  |
| Other | 2089 (4.7) | 1291 (4.8) | 798 (4.5) |  |
| **Diagnoses (ICD-10)** | | | |  |
| Z12: Encounter for screening for malignant neoplasms | 25068 (52.1) | 15094 (52.1) | 9974 (52.1) |  |
| E11: Diabetes mellitus type II | 15277 (31.7) | 9218 (31.8) | 6059 (31.7) |  |
| Z79: Long term (current) drug therapy | 14226 (29.6) | 8588 (29.6) | 5638 (29.5) |  |
| J44: Other chronic obstructive pulmonary disease | 6034 (12.5) | 3690 (12.7) | 2344 (12.2) |  |
| J30: Vasomotor and Allergic Rhinitis | 5757 (12.0) | 3478 (12.0) | 2279 (11.9) |  |
| I48: Atrial fibrillation and flutter | 2803 (5.8) | 1679 (5.8) | 1124 (5.9) |  |
| I50: Heart Failure | 3759 (7.8) | 2296 (7.9) | 1463 (7.6) |  |
| I73: Other peripheral vascular diseases | 2140 (4.4) | 1298 (4.5) | 842 (4.4) |  |
| **Laboratory Abnormalities (Blood)** | | | | |
| Red Blood Cell Count | 18345 (38.1) | 10995 (37.9) | 7350 (38.4) |  |
| Creatinine | 15549 (32.3) | 9364 (32.3) | 6185 (32.3) |  |
| Sodium | 8318 (17.3) | 5049 (17.4) | 3269 (17.1) |  |
| Hematocrit | 5824 (12.1) | 3474 (12.0) | 2350 (12.3) |  |
| Abnormal Albumin | 5789 (12.0) | 3439 (11.9) | 2350 (12.3) |  |
| Alkaline Phosphatase (elevated) | 2329 (4.8) | 1419 (4.9) | 910 (4.8) |  |
| Bicarbonate | 1201 (2.5) | 734 (2.5) | 467 (2.4) |  |
| **Clinical Testing Performed** | | | | |
| Electrocardiogram Prior year | 13425 (27.9) | 8111 (28.0) | 5314 (27.8) |  |
| **Healthcare Utilization** | | | | |
| ED Visit Prior Year | 17304 (36.0) | 10380 (35.8) | 6924 (36.2) |  |

Data reported as n (%) unless otherwise specified. ICD: International Classification of Diseases.
